# Supplementary material for: Tau and spectraplakins promote synapse formation and maintenance through Jun kinase and neuronal trafficking
Source: eLife. 2016 Aug 8;5:e14694. doi: 10.7554/eLife.14694 (PMC4977155; doi:10.7554/eLife.14694)
Supplement: Figure 1—source data 1. — DOI: http://dx.doi.org/10.7554/eLife.14694.004 [file elife-14694-fig1-data1.docx]

**[Figure 1—source data 1](http://elifesciences.org/content/1/e00109v1#SD1-data) Statistics summary**

**Figure 1B Syt puncta**

|  | wt | tau^-/-^ | tau^-/-^ UAS-tau |
| --- | --- | --- | --- |
| Number of values | 679 | 400 | 96 |
|  |  |  |  |
| Minimum | 0.0 | 0.0 | 0.05348 |
| 25% Percentile | 0.4088 | 0.1665 | 0.3209 |
| Median | 0.8743 | 0.4645 | 0.5882 |
| 75% Percentile | 1.402 | 0.8804 | 0.9626 |
| Maximum | 5.351 | 2.694 | 3.369 |
|  |  |  |  |
| Mean | 1.000 | 0.5990 | 0.7921 |
| Std. Deviation | 0.7775 | 0.5381 | 0.6871 |
| Std. Error | 0.02984 | 0.02691 | 0.07012 |
|  |  |  |  |

**Figure 1D Syt puncta**

|  | wt | tau^-/-^ | shot^-/-^ | shot^-/-^ tau^-/-^ |
| --- | --- | --- | --- | --- |
| Number of values | 679 | 400 | 228 | 265 |
|  |  |  |  |  |
| Minimum | 0.0 | 0.0 | 0.0 | 0.0 |
| 25% Percentile | 0.4088 | 0.1665 | 0.2973 | 0.08945 |
| Median | 0.8743 | 0.4645 | 0.6422 | 0.2683 |
| 75% Percentile | 1.402 | 0.8804 | 0.9290 | 0.5574 |
| Maximum | 5.351 | 2.694 | 2.872 | 2.842 |
|  |  |  |  |  |
| Mean | 1.000 | 0.5990 | 0.6729 | 0.3960 |
| Std. Deviation | 0.7775 | 0.5381 | 0.5120 | 0.4390 |
| Std. Error | 0.02984 | 0.02691 | 0.03391 | 0.02697 |

**Figure 1D Brp puncta**

|  | wt | tau^-/-^ | shot^-/-^ | shot^-/-^ tau^-/-^ | |
| --- | --- | --- | --- | --- | --- |
| Number of values | 556 | 251 | 253 | 82 |  |
|  |  |  |  |  |  |
| Minimum | 0.0 | 0.0 | 0.0 | 0.0 |  |
| 25% Percentile | 0.3569 | 0.1191 | 0.0 | 0.0 |  |
| Median | 0.8634 | 0.2977 | 0.3338 | 0.04724 |  |
| 75% Percentile | 1.463 | 0.5954 | 0.8923 | 0.2952 |  |
| Maximum | 5.116 | 2.292 | 4.819 | 1.606 |  |
|  |  |  |  |  |  |
| Mean | 1.000 | 0.4187 | 0.6210 | 0.2229 |  |
| Std. Deviation | 0.8448 | 0.4074 | 0.8162 | 0.3172 |  |
| Std. Error | 0.03583 | 0.02572 | 0.05131 | 0.03503 |  |
