# Supplementary material for: Tau and spectraplakins promote synapse formation and maintenance through Jun kinase and neuronal trafficking
Source: eLife. 2016 Aug 8;5:e14694. doi: 10.7554/eLife.14694 (PMC4977155; doi:10.7554/eLife.14694)
Supplement: Figure 1—figure supplement 2—source data 1. — DOI: http://dx.doi.org/10.7554/eLife.14694.007 [file elife-14694-fig1-figsupp2-data1.docx]

**[Figure 1—suplement 2 source data 1](http://elifesciences.org/content/1/e00109v1#SD1-data) Statistics summary**

**Figure 1-S2 Syt puncta**

| \|  \| wt \| shot-/- \| shot-/-  UAS-shot-GFP \| shot-/- UAS-tau-GFP \| \| --- \| --- \| --- \| --- \| --- \| \| Number of values \| 814 \| 228 \| 444 \| 139 \| \|  \|  \|  \|  \|  \| \| Minimum \| 0.0 \| 0.0 \| 0.0 \| 0.0 \| \| 25% Percentile \| 0.3709 \| 0.2973 \| 0.4574 \| 0.2790 \| \| Median \| 0.8743 \| 0.6422 \| 0.8189 \| 0.7971 \| \| 75% Percentile \| 1.413 \| 0.9290 \| 1.475 \| 1.354 \| \| Maximum \| 5.620 \| 2.872 \| 7.775 \| 4.543 \| \|  \|  \|  \|  \|  \| \| Mean \| 1.000 \| 0.6729 \| 1.136 \| 0.9169 \| \| Std. Deviation \| 0.8069 \| 0.5120 \| 1.103 \| 0.8081 \| \| Std. Error \| 0.02828 \| 0.03391 \| 0.05234 \| 0.06854 \| \|  \|  \|  \|  \|  \| \| Lower 95% CI of mean \| 0.9447 \| 0.6061 \| 1.033 \| 0.7813 \| \| Upper 95% CI of mean \| 1.056 \| 0.7397 \| 1.239 \| 1.052 \| \|  \|  \|  \|  \|  \| \| Sum \| 814.2 \| 153.4 \| 504.5 \| 127.4 \| |  |  |  |
| --- | --- | --- | --- | --- | --- | --- | --- | --- | --- | --- | --- | --- | --- | --- | --- | --- | --- | --- | --- | --- | --- | --- | --- | --- | --- | --- | --- | --- | --- | --- | --- | --- | --- | --- | --- | --- | --- | --- | --- | --- | --- | --- | --- | --- | --- | --- | --- | --- | --- | --- | --- | --- | --- | --- | --- | --- | --- | --- | --- | --- | --- | --- | --- | --- | --- | --- | --- | --- | --- | --- | --- | --- | --- | --- | --- | --- | --- | --- | --- | --- | --- | --- | --- | --- | --- | --- | --- | --- |
|  |  |  |  |
|  |  |  |  |
|  |  |  |  |
| \|  \| wt \| tau-/- \| tau-/-  UAS-shot-GFP \| \| --- \| --- \| --- \| --- \| \| Number of values \| 132 \| 164 \| 172 \| \|  \|  \|  \|  \| \| Minimum \| 0.0 \| 0.0 \| 0.0 \| \| 25% Percentile \| 0.4668 \| 0.05187 \| 0.2075 \| \| Median \| 0.8817 \| 0.3112 \| 0.5670 \| \| 75% Percentile \| 1.387 \| 0.7861 \| 1.127 \| \| Maximum \| 3.831 \| 2.334 \| 5.634 \| \|  \|  \|  \|  \| \| Mean \| 1.000 \| 0.5107 \| 0.7870 \| \| Std. Deviation \| 0.7319 \| 0.5594 \| 0.8140 \| \| Std. Error \| 0.06370 \| 0.04368 \| 0.06207 \| \|  \|  \|  \|  \| \| Lower 95% CI of mean \| 0.8740 \| 0.4244 \| 0.6645 \| \| Upper 95% CI of mean \| 1.126 \| 0.5970 \| 0.9095 \| \|  \|  \|  \|  \| \| Sum \| 132.0 \| 83.76 \| 135.4 \| |  |  |  |
|  |  |  |  |
|  |  |  |  |
|  |  |  |  |
|  |  |  |  |
|  |  |  |  |
|  |  |  |  |
|  |  |  |  |
|  |  |  |  |
