# Supplementary material for: Tau and spectraplakins promote synapse formation and maintenance through Jun kinase and neuronal trafficking
Source: eLife. 2016 Aug 8;5:e14694. doi: 10.7554/eLife.14694 (PMC4977155; doi:10.7554/eLife.14694)
Supplement: Figure 2—source data 1. — DOI: http://dx.doi.org/10.7554/eLife.14694.009 [file elife-14694-fig2-data1.docx]

**[Figure 2—source data 1](http://elifesciences.org/content/1/e00109v1#SD1-data) Statistics summary**

**Figure 2B Syt intensity at embryonic NMJ**

|  | wt | tau^-/-^ | shot^-/-^ | shot^-/-^ tau^-/-^ |
| --- | --- | --- | --- | --- |
| Number of values | 308 | 53 | 90 | 74 |
|  |  |  |  |  |
| Minimum | -0.6897 | -0.03104 | -0.3582 | -0.4759 |
| 25% Percentile | 0.6246 | 0.5018 | 0.2807 | 0.1026 |
| Median | 0.9505 | 0.7617 | 0.6572 | 0.3234 |
| 75% Percentile | 1.292 | 1.234 | 1.039 | 0.8129 |
| Maximum | 3.667 | 2.592 | 4.706 | 1.758 |
|  |  |  |  |  |
| Mean | 1.000 | 0.9235 | 0.7077 | 0.4511 |
| Std. Deviation | 0.5835 | 0.5631 | 0.6679 | 0.5024 |
| Std. Error | 0.03325 | 0.07734 | 0.07040 | 0.05841 |
