# Supplementary material for: Tau and spectraplakins promote synapse formation and maintenance through Jun kinase and neuronal trafficking
Source: eLife. 2016 Aug 8;5:e14694. doi: 10.7554/eLife.14694 (PMC4977155; doi:10.7554/eLife.14694)
Supplement: Figure 3—source data 1. — DOI: http://dx.doi.org/10.7554/eLife.14694.012 [file elife-14694-fig3-data1.docx]

**[Figure 3—source data 1](http://elifesciences.org/content/1/e00109v1#SD1-data) Statistics summary**

**Figure 3B Syt puncta**

|  | wt 3d | tauRNAi shotRNAi 3d | wt 18d | tauRNAi shotRNAi 18d | wt 26d | tauRNAi shotRNAi 26 d |
| --- | --- | --- | --- | --- | --- | --- |
| Number of values | 269 | 171 | 210 | 252 | 316 | 280 |
|  |  |  |  |  |  |  |
| Minimum | 0.0 | 0.0 | 0.0 | 0.0 | 0.0 | 0.0 |
| 25% Percentile | 0.1114 | 0.04225 | 0.0 | 0.1230 | 0.0 | 0.0 |
| Median | 0.8873 | 0.6718 | 0.6151 | 0.5553 | 0.1771 | 0.06901 |
| 75% Percentile | 1.632 | 1.824 | 1.563 | 1.555 | 1.242 | 0.3541 |
| Maximum | 4.079 | 4.511 | 8.119 | 9.350 | 11.51 | 5.666 |
|  |  |  |  |  |  |  |
| Mean | 0.9963 | 1.056 | 1.000 | 1.042 | 1.000 | 0.4145 |
| Std. Deviation | 0.8689 | 1.078 | 1.334 | 1.344 | 1.856 | 0.8598 |
| Std. Error | 0.05298 | 0.08242 | 0.09207 | 0.08465 | 0.1044 | 0.05138 |

**Figure 3D Normalised number of synapses in old flies**

|  | wt | tauRNAi | shotRNAi | shotRNAi+tauRNAi |
| --- | --- | --- | --- | --- |
| Number of values | 25 | 35 | 30 | 61 |
|  |  |  |  |  |
| Minimum | 0.5300 | 0.1700 | 0.1300 | 0.0100 |
| 25% Percentile | 0.9350 | 0.8900 | 0.9625 | 0.2600 |
| Median | 1.260 | 1.280 | 1.160 | 0.4505 |
| 75% Percentile | 2.455 | 1.750 | 1.493 | 0.7200 |
| Maximum | 4.350 | 2.720 | 2.400 | 3.410 |
|  |  |  |  |  |
| Mean | 1.692 | 1.319 | 1.192 | 0.5710 |
| Std. Deviation | 1.042 | 0.6080 | 0.4536 | 0.5224 |
| Std. Error | 0.2083 | 0.1028 | 0.08281 | 0.06689 |

**Figure 3F Average branch number**

|  | Young control | Young tauRNAi shotRNAi | Old control | Old tauRNAi shotRNAi |
| --- | --- | --- | --- | --- |
| Number of values | 41 | 35 | 36 | 26 |
|  |  |  |  |  |
| Minimum | 3.000 | 2.000 | 3.000 | 3.000 |
| 25% Percentile | 5.000 | 4.000 | 5.000 | 4.750 |
| Median | 5.000 | 5.000 | 6.000 | 6.000 |
| 75% Percentile | 6.000 | 6.000 | 6.000 | 6.250 |
| Maximum | 9.000 | 9.000 | 8.000 | 8.000 |
|  |  |  |  |  |
| Mean | 5.537 | 5.000 | 5.556 | 5.577 |
| Std. Deviation | 1.343 | 1.372 | 1.206 | 1.301 |
| Std. Error | 0.2098 | 0.2319 | 0.2010 | 0.2552 |
