# Supplementary material for: Tau and spectraplakins promote synapse formation and maintenance through Jun kinase and neuronal trafficking
Source: eLife. 2016 Aug 8;5:e14694. doi: 10.7554/eLife.14694 (PMC4977155; doi:10.7554/eLife.14694)
Supplement: Figure 3—figure supplement 1—source data 1. — DOI: http://dx.doi.org/10.7554/eLife.14694.014 [file elife-14694-fig3-figsupp1-data1.docx]

[**Figure 3—supplement 1 source data 1**](http://elifesciences.org/content/1/e00109v1#SD1-data) **Statistics summary**

**Figure 3-S1B Mean intensity of anti tau anti shot**

| \|  \| wt  3d  tau \| shot-tau RNAi 3d  tau \| tau-/-  3d  tau \| wt  3d  shot \| tau-shot RNAi 3d  shot \| shot-/-  3d  shot \| wt  25d  tau \| tau-shot RNAi 25d  tau \| wt  25d  shot \| tau-shot RNAi 25d  shot \| \| --- \| --- \| --- \| --- \| --- \| --- \| --- \| --- \| --- \| --- \| --- \| \| Number of values \| 32 \| 34 \| 76 \| 32 \| 34 \| 98 \| 39 \| 31 \| 39 \| 31 \| \|  \|  \|  \|  \|  \|  \|  \|  \|  \|  \|  \| \| Minimum \| 0.2264 \| 0.07422 \| 0.04058 \| 0.3747 \| 0.6088 \| 0.3487 \| 0.09661 \| 0.06757 \| 0.5433 \| 0.3311 \| \| 25% Percentile \| 0.6432 \| 0.09564 \| 0.05121 \| 0.7978 \| 0.7675 \| 0.4327 \| 0.6497 \| 0.08146 \| 0.7006 \| 0.3974 \| \| Median \| 0.9605 \| 0.1237 \| 0.05746 \| 1.026 \| 0.9589 \| 0.5295 \| 1.067 \| 0.09614 \| 1.079 \| 0.4893 \| \| 75% Percentile \| 1.258 \| 0.1614 \| 0.06436 \| 1.133 \| 1.219 \| 0.6103 \| 1.350 \| 0.1144 \| 1.206 \| 0.5606 \| \| Maximum \| 2.424 \| 1.302 \| 0.5123 \| 1.567 \| 1.807 \| 1.174 \| 1.837 \| 0.6073 \| 1.599 \| 0.9532 \| \|  \|  \|  \|  \|  \|  \|  \|  \|  \|  \|  \| \| Mean \| 1.000 \| 0.1843 \| 0.06670 \| 1.000 \| 1.042 \| 0.5408 \| 1.000 \| 0.1141 \| 1.000 \| 0.5143 \| \| Std. Deviation \| 0.4811 \| 0.2335 \| 0.05485 \| 0.2415 \| 0.3294 \| 0.1432 \| 0.4495 \| 0.09389 \| 0.2937 \| 0.1475 \| \| Std. Error \| 0.08506 \| 0.04004 \| 0.006291 \| 0.04270 \| 0.05649 \| 0.01447 \| 0.07198 \| 0.01686 \| 0.04703 \| 0.02649 \| \|  \|  \|  \|  \|  \|  \|  \|  \|  \|  \|  \| \| Lower 95% CI of mean \| 0.8265 \| 0.1028 \| 0.05417 \| 0.9129 \| 0.9272 \| 0.5121 \| 0.8543 \| 0.07970 \| 0.9048 \| 0.4602 \| \| Upper 95% CI of mean \| 1.173 \| 0.2657 \| 0.07923 \| 1.087 \| 1.157 \| 0.5695 \| 1.146 \| 0.1486 \| 1.095 \| 0.5684 \| \|  \|  \|  \|  \|  \|  \|  \|  \|  \|  \|  \| \| Sum \| 32.00 \| 6.265 \| 5.069 \| 32.00 \| 35.43 \| 53.00 \| 39.00 \| 3.538 \| 39.00 \| 15.94 \| |  |  |  |
| --- | --- | --- | --- | --- | --- | --- | --- | --- | --- | --- | --- | --- | --- | --- | --- | --- | --- | --- | --- | --- | --- | --- | --- | --- | --- | --- | --- | --- | --- | --- | --- | --- | --- | --- | --- | --- | --- | --- | --- | --- | --- | --- | --- | --- | --- | --- | --- | --- | --- | --- | --- | --- | --- | --- | --- | --- | --- | --- | --- | --- | --- | --- | --- | --- | --- | --- | --- | --- | --- | --- | --- | --- | --- | --- | --- | --- | --- | --- | --- | --- | --- | --- | --- | --- | --- | --- | --- | --- | --- | --- | --- | --- | --- | --- | --- | --- | --- | --- | --- | --- | --- | --- | --- | --- | --- | --- | --- | --- | --- | --- | --- | --- | --- | --- | --- | --- | --- | --- | --- | --- | --- | --- | --- | --- | --- | --- | --- | --- | --- | --- | --- | --- | --- | --- | --- | --- | --- | --- | --- | --- | --- | --- | --- | --- | --- | --- | --- | --- | --- | --- | --- | --- | --- | --- | --- | --- | --- | --- | --- | --- | --- | --- | --- | --- | --- | --- | --- | --- | --- | --- | --- | --- | --- | --- | --- | --- | --- | --- | --- | --- | --- | --- | --- | --- | --- | --- | --- | --- | --- | --- |
|  |  |  |  |
|  |  |  |  |
|  |  |  |  |
|  |  |  |  |
|  |  |  |  |
|  |  |  |  |
