# Supplementary material for: Tau and spectraplakins promote synapse formation and maintenance through Jun kinase and neuronal trafficking
Source: eLife. 2016 Aug 8;5:e14694. doi: 10.7554/eLife.14694 (PMC4977155; doi:10.7554/eLife.14694)
Supplement: Figure 3—figure supplement 4—source data 1. — DOI: http://dx.doi.org/10.7554/eLife.14694.018 [file elife-14694-fig3-figsupp4-data1.docx]

**[Figure 3—supplement 4 source data 1](http://elifesciences.org/content/1/e00109v1#SD1-data) Statistics summary**

**Figure 3-S4 axon length**

|  |  |  |  |
| --- | --- | --- | --- |
|  |  |  |  |
|  |  |  |  |
|  |  |  |  |
| \|  \| control 3days \| shot-tau RNAi  3days \|  \| control 18days \| shot-tau RNAi 18days \|  \| control 26days \| shot-tau RNAi 26days \| \| --- \| --- \| --- \| --- \| --- \| --- \| --- \| --- \| --- \| \| Number of values \| 130 \| 116 \|  \| 99 \| 100 \|  \| 111 \| 97 \| \|  \|  \|  \|  \|  \|  \|  \|  \|  \| \| Minimum \| 0.2313 \| 0.3421 \|  \| 0.3824 \| 0.2350 \|  \| 0.2940 \| 0.3710 \| \| 25% Percentile \| 0.5968 \| 0.7175 \|  \| 0.6783 \| 0.6185 \|  \| 0.7040 \| 0.7190 \| \| Median \| 0.9313 \| 0.9546 \|  \| 0.9042 \| 0.8307 \|  \| 0.9230 \| 0.9440 \| \| 75% Percentile \| 1.322 \| 1.242 \|  \| 1.222 \| 1.066 \|  \| 1.214 \| 1.282 \| \| Maximum \| 2.483 \| 3.784 \|  \| 2.962 \| 2.026 \|  \| 2.827 \| 3.221 \| \|  \|  \|  \|  \|  \|  \|  \|  \|  \| \| Mean \| 1.007 \| 1.075 \|  \| 1.000 \| 0.8853 \|  \| 1.000 \| 1.082 \| \| Std. Deviation \| 0.4949 \| 0.5922 \|  \| 0.4560 \| 0.3491 \|  \| 0.4305 \| 0.5340 \| \| Std. Error \| 0.04341 \| 0.05499 \|  \| 0.04583 \| 0.03491 \|  \| 0.04086 \| 0.05422 \| \|  \|  \|  \|  \|  \|  \|  \|  \|  \| \| Lower 95% CI of mean \| 0.9214 \| 0.9658 \|  \| 0.9093 \| 0.8160 \|  \| 0.9191 \| 0.9743 \| \| Upper 95% CI of mean \| 1.093 \| 1.184 \|  \| 1.091 \| 0.9546 \|  \| 1.081 \| 1.190 \| \|  \|  \|  \|  \|  \|  \|  \|  \|  \| \| Sum \| 130.9 \| 124.7 \|  \| 99.02 \| 88.53 \|  \| 111.0 \| 104.9 \| |  |  |  |
|  |  |  |  |
|  |  |  |  |
|  |  |  |  |
| **Figure 3-S4 number branches** |  |  |  |
|  |  |  |  |
|  |  |  |  |
|  |  |  |  |
| \|  \| control 3days \| shot-tau RNAi 3days \|  \| control 18days \| shot-tau RNAi 18days \|  \| control 26days \| shot-tau RNAi 26days \| \| --- \| --- \| --- \| --- \| --- \| --- \| --- \| --- \| --- \| \| Number of values \| 130 \| 115 \|  \| 99 \| 100 \|  \| 114 \| 107 \| \|  \|  \|  \|  \|  \|  \|  \|  \|  \| \| Minimum \| 0.0 \| 0.0 \|  \| 0.0 \| 0.0 \|  \| 0.0 \| 0.0 \| \| 25% Percentile \| 0.4815 \| 0.5010 \|  \| 0.5390 \| 0.6470 \|  \| 0.4068 \| 0.5972 \| \| Median \| 0.8770 \| 0.8770 \|  \| 0.8333 \| 1.078 \|  \| 0.8547 \| 0.9153 \| \| 75% Percentile \| 1.504 \| 1.378 \|  \| 1.401 \| 1.667 \|  \| 1.344 \| 1.424 \| \| Maximum \| 2.882 \| 2.821 \|  \| 3.556 \| 4.405 \|  \| 5.390 \| 3.356 \| \|  \|  \|  \|  \|  \|  \|  \|  \|  \| \| Mean \| 0.9969 \| 0.9700 \|  \| 1.000 \| 1.176 \|  \| 0.9737 \| 1.117 \| \| Std. Deviation \| 0.6226 \| 0.6470 \|  \| 0.6248 \| 0.7371 \|  \| 0.8254 \| 0.7658 \| \| Std. Error \| 0.05460 \| 0.06034 \|  \| 0.06280 \| 0.07371 \|  \| 0.07731 \| 0.07403 \| \|  \|  \|  \|  \|  \|  \|  \|  \|  \| \| Lower 95% CI of mean \| 0.8888 \| 0.8504 \|  \| 0.8755 \| 1.030 \|  \| 0.8206 \| 0.9705 \| \| Upper 95% CI of mean \| 1.105 \| 1.089 \|  \| 1.125 \| 1.323 \|  \| 1.127 \| 1.264 \| \|  \|  \|  \|  \|  \|  \|  \|  \|  \| \| Sum \| 129.6 \| 111.5 \|  \| 99.01 \| 117.6 \|  \| 111.0 \| 119.5 \| |  |  |  |
