# Supplementary material for: Tau and spectraplakins promote synapse formation and maintenance through Jun kinase and neuronal trafficking
Source: eLife. 2016 Aug 8;5:e14694. doi: 10.7554/eLife.14694 (PMC4977155; doi:10.7554/eLife.14694)
Supplement: Figure 4—source data 1. — DOI: http://dx.doi.org/10.7554/eLife.14694.020 [file elife-14694-fig4-data1.docx]

**[Figure 4—source data 1](http://elifesciences.org/content/1/e00109v1#SD1-data) Statistics summary**

**Figure 4B Syb. puncta**

|  | wt | shot^-/-^tau^-/-^ |
| --- | --- | --- |
| Number of values | 45 | 74 |
|  |  |  |
| Minimum | 0.0 | 0.0 |
| 25% Percentile | 0.4452 | 0.0 |
| Median | 0.8219 | 0.1370 |
| 75% Percentile | 1.473 | 0.4795 |
| Maximum | 2.808 | 2.055 |
|  |  |  |
| Mean | 1.000 | 0.3138 |
| Std. Deviation | 0.7623 | 0.4405 |
| Std. Error | 0.1136 | 0.05121 |

**Figure 4B Syt. puncta**

|  | wt | shot-/-tau-/- |
| --- | --- | --- |
| Number of values | 78 | 82 |
|  |  |  |
| Minimum | 0.0 | 0.0 |
| 25% Percentile | 0.1714 | 0.0 |
| Median | 0.5143 | 0.1714 |
| 75% Percentile | 1.414 | 0.5143 |
| Maximum | 5.657 | 2.743 |
|  |  |  |
| Mean | 1.000 | 0.3367 |
| Std. Deviation | 1.174 | 0.5344 |
| Std. Error | 0.1329 | 0.05866 |

**Figure 4C n. vesicles in axon**

|  | control | shot^-/-^ tau^-/-^ |
| --- | --- | --- |
| Number of values | 9 | 12 |
|  |  |  |
| Minimum | 10.00 | 0.0 |
| 25% Percentile | 12.50 | 1.500 |
| Median | 16.00 | 5.500 |
| 75% Percentile | 33.50 | 9.000 |
| Maximum | 44.00 | 37.00 |
|  |  |  |
| Mean | 23.11 | 8.500 |
| Std. Deviation | 12.45 | 10.45 |
| Std. Error | 4.151 | 3.016 |
|  |  |  |

**Figure 4C n. vesicles in soma**

|  | control | shot^-/-^tau^-/-^ |
| --- | --- | --- |
| Number of values | 9 | 12 |
|  |  |  |
| Minimum | 11.70 | 25.00 |
| 25% Percentile | 25.00 | 37.25 |
| Median | 28.00 | 78.10 |
| 75% Percentile | 64.00 | 90.14 |
| Maximum | 75.00 | 100.0 |
|  |  |  |
| Mean | 41.88 | 66.69 |
| Std. Deviation | 23.26 | 27.01 |
| Std. Error | 7.752 | 7.798 |

**Figure 4C velocity**

| \|  \| wt anterograde \| shot-/- tau-/- anterograde \| wt retrograde \| shot-/- tau-/- retrograde \| \| --- \| --- \| --- \| --- \| --- \| \| Number of values \| 1052 \| 966 \| 1075 \| 916 \| \|  \|  \|  \|  \|  \| \| Minimum \| 0.0645 \| 0.0645 \| -3.142 \| -4.967 \| \| 25% Percentile \| 0.1440 \| 0.1825 \| -0.5840 \| -0.6385 \| \| Median \| 0.3225 \| 0.3290 \| -0.2885 \| -0.2885 \| \| 75% Percentile \| 0.5945 \| 0.6855 \| -0.1440 \| -0.1290 \| \| Maximum \| 6.412 \| 3.821 \| -0.0645 \| -0.0645 \| \|  \|  \|  \|  \|  \| \| Mean \| 0.4521 \| 0.5087 \| -0.4468 \| -0.4821 \| \| Std. Deviation \| 0.4769 \| 0.5084 \| 0.4495 \| 0.5504 \| \| Std. Error \| 0.01470 \| 0.01636 \| 0.01371 \| 0.01818 \| |  |  |
| --- | --- | --- | --- | --- | --- | --- | --- | --- | --- | --- | --- | --- | --- | --- | --- | --- | --- | --- | --- | --- | --- | --- | --- | --- | --- | --- | --- | --- | --- | --- | --- | --- | --- | --- | --- | --- | --- | --- | --- | --- | --- | --- | --- | --- | --- | --- | --- | --- | --- | --- | --- | --- | --- | --- | --- | --- | --- | --- | --- | --- | --- | --- |

**Figure 4D Syt. intensity in soma**

|  | WT | shot^-/-^ tau^-/-^ |
| --- | --- | --- |
| Number of values | 111 | 102 |
|  |  |  |
| Minimum | 0.3276 | 0.4388 |
| 25% Percentile | 0.6752 | 1.077 |
| Median | 0.9191 | 1.458 |
| 75% Percentile | 1.170 | 1.822 |
| Maximum | 2.415 | 2.440 |
|  |  |  |
| Mean | 1.000 | 1.441 |
| Std. Deviation | 0.4172 | 0.4475 |
| Std. Error | 0.03960 | 0.04431 |
