# Supplementary material for: Tau and spectraplakins promote synapse formation and maintenance through Jun kinase and neuronal trafficking
Source: eLife. 2016 Aug 8;5:e14694. doi: 10.7554/eLife.14694 (PMC4977155; doi:10.7554/eLife.14694)
Supplement: Figure 5—source data 1. — DOI: http://dx.doi.org/10.7554/eLife.14694.022 [file elife-14694-fig5-data1.docx]

| **[Figure 5—source data 1](http://elifesciences.org/content/1/e00109v1#SD1-data) Statistics summary**  **Figure 5A genetic interaction Unc-104**   \|  \| wt \| shot3^+/-^tau^+/-^ \| unc-104^+/-^ \| shot3^+/-^tau^+/-^unc104^+/-^ \| unc104^-/-^ \| \| --- \| --- \| --- \| --- \| --- \| --- \| \| Number of values \| 350 \| 222 \| 201 \| 55 \| 71 \| \|  \|  \|  \|  \|  \|  \| \| Minimum \| 0.0 \| 0.0 \| 0.0 \| 0.0 \| 0.0 \| \| 25% Percentile \| 0.4033 \| 0.2510 \| 0.04184 \| 0.1452 \| 0.0 \| \| Median \| 0.9355 \| 0.7181 \| 0.8368 \| 0.5589 \| 0.04749 \| \| 75% Percentile \| 1.492 \| 1.256 \| 1.472 \| 0.8715 \| 0.2713 \| \| Maximum \| 3.527 \| 3.849 \| 7.053 \| 2.150 \| 2.849 \| \|  \|  \|  \|  \|  \|  \| \| Mean \| 1.003 \| 0.8595 \| 1.006 \| 0.5959 \| 0.2532 \| \| Std. Deviation \| 0.7743 \| 0.7749 \| 1.080 \| 0.5047 \| 0.4782 \| \| Std. Error \| 0.04139 \| 0.05201 \| 0.07621 \| 0.06805 \| 0.05675 \|   **Figure 5B Unc-104 shot^-/-^tau^-/-^ rescue**   \|  \| wt \| shot^-/-^tau^-/-^  UAS-104 \| \| --- \| --- \| --- \| \| Number of values \| 161 \| 158 \| \|  \|  \|  \| \| Minimum \| 0.0 \| 0.0 \| \| 25% Percentile \| 0.05594 \| 0.08247 \| \| Median \| 0.7835 \| 0.5889 \| \| 75% Percentile \| 1.554 \| 1.325 \| \| Maximum \| 5.856 \| 5.691 \| \|  \|  \|  \| \| Mean \| 0.9939 \| 0.9162 \| \| Std. Deviation \| 1.041 \| 1.008 \| \| Std. Error \| 0.08202 \| 0.08015 \|   **Figure 5D Unc-104 ratio distal axon/soma** | | | |
| --- | --- | --- | --- | --- | --- | --- | --- | --- | --- | --- | --- | --- | --- | --- | --- | --- | --- | --- | --- | --- | --- | --- | --- | --- | --- | --- | --- | --- | --- | --- | --- | --- | --- | --- | --- | --- | --- | --- | --- | --- | --- | --- | --- | --- | --- | --- | --- | --- | --- | --- | --- | --- | --- | --- | --- | --- | --- | --- | --- | --- | --- | --- | --- | --- | --- | --- | --- | --- | --- | --- | --- | --- | --- | --- | --- | --- | --- | --- | --- | --- | --- | --- | --- | --- | --- | --- | --- | --- | --- | --- | --- | --- | --- | --- | --- | --- | --- | --- | --- | --- | --- | --- | --- | --- | --- | --- | --- | --- | --- | --- | --- |
|  |  |  |  |
|  | WT | shot^-/-^ tau^-/-^ |  |
| Number of values | 72 | 45 |  |
|  |  |  |  |
| Minimum | 0.3467 | 0.2635 |  |
| 25% Percentile | 0.5570 | 0.3600 |  |
| Median | 0.7974 | 0.4436 |  |
| 75% Percentile | 1.145 | 0.6810 |  |
| Maximum | 3.932 | 1.419 |  |
|  |  |  |  |
| Mean | 0.9999 | 0.5489 |  |
| Std. Deviation | 0.6560 | 0.2689 |  |
| Std. Error | 0.07731 | 0.04009 |  |
