# Supplementary material for: Tau and spectraplakins promote synapse formation and maintenance through Jun kinase and neuronal trafficking
Source: eLife. 2016 Aug 8;5:e14694. doi: 10.7554/eLife.14694 (PMC4977155; doi:10.7554/eLife.14694)
Supplement: Figure 5—figure supplement 1—source data 1. — DOI: http://dx.doi.org/10.7554/eLife.14694.024 [file elife-14694-fig5-figsupp1-data1.docx]

**[Figure 5—supplement 1 source data 1](http://elifesciences.org/content/1/e00109v1#SD1-data) Statistics summary**

**Figure 5-S1 Ageing synapse index (old/mean young)**

|  |  |  |  |
| --- | --- | --- | --- |
|  |  |  |  |
|  |  |  |  |
| \|  \| cont \| tau-shot RNAi \| tau-shot RNAi  UAS-unc104 \| \| --- \| --- \| --- \| --- \| \| Number of values \| 23 \| 40 \| 64 \| \|  \|  \|  \|  \| \| Minimum \| 0.9931 \| 0.08621 \| 0.4029 \| \| 25% Percentile \| 1.473 \| 0.5439 \| 1.561 \| \| Median \| 1.936 \| 1.380 \| 2.754 \| \| 75% Percentile \| 2.716 \| 2.152 \| 3.542 \| \| Maximum \| 4.430 \| 2.718 \| 5.151 \| \|  \|  \|  \|  \| \| Mean \| 2.209 \| 1.329 \| 2.627 \| \| Std. Deviation \| 0.9941 \| 0.8155 \| 1.237 \| \| Std. Error \| 0.2073 \| 0.1289 \| 0.1547 \| \|  \|  \|  \|  \| \| Lower 95% CI of mean \| 1.779 \| 1.068 \| 2.318 \| \| Upper 95% CI of mean \| 2.639 \| 1.590 \| 2.936 \| \|  \|  \|  \|  \| \| Sum \| 50.80 \| 53.16 \| 168.1 \| |  |  |  |
|  |  |  |  |
|  |  |  |  |
|  |  |  |  |
|  |  |  |  |
