# Supplementary material for: Tau and spectraplakins promote synapse formation and maintenance through Jun kinase and neuronal trafficking
Source: eLife. 2016 Aug 8;5:e14694. doi: 10.7554/eLife.14694 (PMC4977155; doi:10.7554/eLife.14694)
Supplement: Figure 6—source data 1. — DOI: http://dx.doi.org/10.7554/eLife.14694.026 [file elife-14694-fig6-data1.docx]

[**Figure 6—source data 1**](http://elifesciences.org/content/1/e00109v1#SD1-data) **Statistics summary**

**Figure 6C nocodazole**

|  | wt | shot-/- | tau-/- | tau-/- uas tau | shot-/-tau-/- |
| --- | --- | --- | --- | --- | --- |
| Number of values | 43 | 46 | 85 | 51 | 49 |
|  |  |  |  |  |  |
| Minimum | 0.0 | 0.0 | 0.0 | 0.0 | 0.0 |
| 25% Percentile | 0.0 | 0.0 | 0.0 | 0.0 | 1.000 |
| Median | 0.0 | 1.000 | 0.0 | 0.0 | 3.000 |
| 75% Percentile | 0.0 | 1.000 | 1.000 | 1.000 | 4.000 |
| Maximum | 0.0 | 4.000 | 8.000 | 2.000 | 6.000 |
|  |  |  |  |  |  |
| Mean | 0.0 | 0.7826 | 0.9529 | 0.3333 | 2.531 |
| Std. Deviation | 0.0 | 0.9168 | 1.405 | 0.5538 | 1.733 |
| Std. Error | 0.0 | 0.1352 | 0.1524 | 0.07754 | 0.2476 |

**Figure 6E Epo.B treatment**

|  | wt DMSO | shot-/-tau-/- DMSO | wt Epo.B | shot-/-tau-/- Epo.B |
| --- | --- | --- | --- | --- |
| Number of values | 77 | 137 | 106 | 86 |
|  |  |  |  |  |
| Minimum | 0.1981 | 0.04436 | 0.1450 | 0.2123 |
| 25% Percentile | 0.4645 | 0.3568 | 0.8057 | 0.6072 |
| Median | 0.7771 | 0.5761 | 1.064 | 0.9626 |
| 75% Percentile | 1.297 | 0.8444 | 1.504 | 1.306 |
| Maximum | 3.266 | 1.858 | 2.187 | 2.815 |
|  |  |  |  |  |
| Mean | 0.9888 | 0.6366 | 1.137 | 1.045 |
| Std. Deviation | 0.6859 | 0.3669 | 0.4895 | 0.5561 |
| Std. Error | 0.07817 | 0.03135 | 0.04755 | 0.05997 |
