# Supplementary material for: Tau and spectraplakins promote synapse formation and maintenance through Jun kinase and neuronal trafficking
Source: eLife. 2016 Aug 8;5:e14694. doi: 10.7554/eLife.14694 (PMC4977155; doi:10.7554/eLife.14694)
Supplement: Figure 6—figure supplement 1—source data 1. — DOI: http://dx.doi.org/10.7554/eLife.14694.028 [file elife-14694-fig6-figsupp1-data1.docx]

**[Figure 6—supplement 1 source data 1](http://elifesciences.org/content/1/e00109v1#SD1-data) Statistics summary**

**Figure 6-S1 Intensity of JNK-P at axonal tips after treatment with epothilone B**

|  |  |  |  |
| --- | --- | --- | --- |
| \|  \| wt DMSO \| Shot-/-Tau-/- DMSO \| WT EpoB \| Shot-/-Tau-/- EpoB \| \| --- \| --- \| --- \| --- \| --- \| \| Number of values \| 55 \| 69 \| 59 \| 74 \| \|  \|  \|  \|  \|  \| \| Minimum \| 0.2725 \| 0.1765 \| 0.4546 \| 0.2365 \| \| 25% Percentile \| 0.8135 \| 0.3470 \| 0.7632 \| 0.5439 \| \| Median \| 0.9827 \| 0.4750 \| 0.9642 \| 0.7713 \| \| 75% Percentile \| 1.192 \| 0.6399 \| 1.234 \| 1.013 \| \| Maximum \| 2.358 \| 1.084 \| 1.525 \| 1.402 \| \|  \|  \|  \|  \|  \| \| Mean \| 1.000 \| 0.5143 \| 0.9999 \| 0.7770 \| \| Std. Deviation \| 0.3045 \| 0.2147 \| 0.2850 \| 0.3038 \| \| Std. Error \| 0.04105 \| 0.02585 \| 0.03711 \| 0.03532 \| \|  \|  \|  \|  \|  \| \| Lower 95% CI of mean \| 0.9178 \| 0.4628 \| 0.9256 \| 0.7066 \| \| Upper 95% CI of mean \| 1.082 \| 0.5659 \| 1.074 \| 0.8474 \| \|  \|  \|  \|  \|  \| \| Sum \| 55.00 \| 35.49 \| 58.99 \| 57.50 \| |  |  |  |
|  |  |  |  |
|  |  |  |  |
|  |  |  |  |
|  |  |  |  |
|  |  |  |  |
|  |  |  |  |
