# Supplementary material for: Tau and spectraplakins promote synapse formation and maintenance through Jun kinase and neuronal trafficking
Source: eLife. 2016 Aug 8;5:e14694. doi: 10.7554/eLife.14694 (PMC4977155; doi:10.7554/eLife.14694)
Supplement: Figure 8—source data 1. — DOI: http://dx.doi.org/10.7554/eLife.14694.032 [file elife-14694-fig8-data1.docx]

**[Figure 8—source data 1](http://elifesciences.org/content/1/e00109v1#SD1-data) Statistics summary**

**Figure 8B JNK pathway**

|  | wt | | tau-/- | | shot-/-tau-/- | | uas Hep-ac | | tau-/- wnd2-/- | | tau-/- uasPuc | | tau-/-kay-/- | | shot-/-tau-/-wnd-/- | | wnd-/- | | uas Puc | kay-/- |
| --- | --- | --- | --- | --- | --- | --- | --- | --- | --- | --- | --- | --- | --- | --- | --- | --- | --- | --- | --- | --- |
| Number of values | | 1322 | | 400 | | 265 | | 178 | | 255 | | 323 | | 174 | | 296 | | 382 | 342 | 144 |
|  | |  | |  | |  | |  | |  | |  | |  | |  | |  |  |  |
| Minimum | | 0.0 | | 0.0 | | 0.0 | | 0.0 | | 0.0 | | 0.0 | | 0.0 | | 0.0 | | 0.0 | 0.0 | 0.0 |
| 25% Percentile | | 0.3357 | | 0.1665 | | 0.08945 | | 0.1176 | | 0.3531 | | 0.2353 | | 0.05181 | | 0.3521 | | 0.5755 | 0.3906 | 0.5099 |
| Median | | 0.9178 | | 0.4645 | | 0.2683 | | 0.2943 | | 0.8240 | | 0.5886 | | 0.2073 | | 0.7042 | | 0.8444 | 0.7674 | 1.020 |
| 75% Percentile | | 1.471 | | 0.8804 | | 0.5574 | | 0.6471 | | 1.353 | | 1.302 | | 0.4145 | | 1.320 | | 1.199 | 1.291 | 1.632 |
| Maximum | | 8.550 | | 2.694 | | 2.842 | | 2.413 | | 4.941 | | 4.882 | | 1.606 | | 4.332 | | 3.597 | 4.685 | 3.977 |
|  | |  | |  | |  | |  | |  | |  | |  | |  | |  |  |  |
| Mean | | 1.000 | | 0.5990 | | 0.3960 | | 0.4304 | | 0.9392 | | 0.8489 | | 0.2768 | | 0.9062 | | 0.9314 | 0.9262 | 1.082 |
| Std. Deviation | | 0.8240 | | 0.5381 | | 0.4390 | | 0.4232 | | 0.7531 | | 0.8212 | | 0.3135 | | 0.7627 | | 0.5771 | 0.7769 | 0.8548 |
| Std. Error | | 0.02266 | | 0.02691 | | 0.02697 | | 0.03172 | | 0.04716 | | 0.04569 | | 0.02370 | | 0.04433 | | 0.02953 | 0.04201 | 0.07123 |

**Figure 8D** NMJ **rescue with wnd**

|  | wt | shot-/- tau-/- | shot-/-tau-/-wnd-/- |
| --- | --- | --- | --- |
| Number of values | 131 | 74 | 74 |
|  |  |  |  |
| Minimum | 0.2440 | -0.4759 | 0.06648 |
| 25% Percentile | 0.6484 | 0.1026 | 0.4546 |
| Median | 0.9507 | 0.3234 | 0.8139 |
| 75% Percentile | 1.239 | 0.8129 | 1.107 |
| Maximum | 3.669 | 1.758 | 2.032 |
|  |  |  |  |
| Mean | 1.006 | 0.4511 | 0.8349 |
| Std. Deviation | 0.4956 | 0.5024 | 0.4763 |
| Std. Error | 0.04330 | 0.05841 | 0.05537 |

**Figure 8F** Ageing synapse index

|  | controle | Tau^RNAi^ Shot^RNAi^ | Tau^RNAi^ Shot^RNAi^ UAS-BSKDN |
| --- | --- | --- | --- |
| Number of values | 23 | 40 | 46 |
|  |  |  |  |
| Minimum | 0.9931 | 0.08621 | 0.3677 |
| 25% Percentile | 1.473 | 0.5439 | 1.501 |
| Median | 1.936 | 1.380 | 2.778 |
| 75% Percentile | 2.716 | 2.152 | 4.062 |
| Maximum | 4.430 | 2.718 | 5.831 |
|  |  |  |  |
| Mean | 2.209 | 1.329 | 2.803 |
| Std. Deviation | 0.9941 | 0.8155 | 1.483 |
| Std. Error | 0.2073 | 0.1289 | 0.2187 |
